# A C terminus–dependent conformational change is required for HDAC3 activation by nuclear receptor corepressors

**DOI:** 10.1016/j.jbc.2021.101192

**Published:** 2021-09-11

**Authors:** Jian Li, Chun Guo, Christopher Rood, Jinsong Zhang

**Affiliations:** Department of Pharmacology & Physiology, Saint Louis University School of Medicine, St Louis, Missouri, USA

**Keywords:** HDAC3, conformational change, C terminus, deacetylase-activation domain, DAD, deacetylase-activation domain, FL, full-length, HDAC, histone deacetylase, HDLP, a histone deacetylase–like protein, IP4, inositol tetraphosphate, NCoR, nuclear receptor corepressor, SANT, SWI3, ADA2, N-CoR or SMRT, and TFIIIB homology domain, SMRT, silencing mediator of retinoic acid and thyroid hormone receptor, TSA, trichostatin A

## Abstract

Histone deacetylase 3 (HDAC3) plays an important role in signal-dependent transcription and is dysregulated in diseases such as cancer. Previous studies have shown that the function of HDAC3 requires an activation step, which is mediated by the interactions of HDAC3 with the deacetylase-activation domain (DAD) of nuclear receptor corepressors and inositol tetraphosphate (IP4). However, the role of the unique HDAC3 C-terminal region in HDAC3 activation is elusive. Here multiple biochemical, structural, and functional studies show that HDAC3 activation requires a priming step mediated by the C terminus to remodel HDAC3 conformation. We show that multiple C-terminal mutations prevent HDAC3 activation by preventing this C terminus–dependent conformational change. Mechanistically, we demonstrate that the C terminus–mediated function in altering HDAC3 conformation is required for proper complex formation of HDAC3 with DAD and IP4 by allowing HDAC3 to undergo IP4-dependent interaction with DAD. Remarkably, we found that this C terminus function is conformation dependent, being necessary for HDAC3 activation prior to but not after the conformational change. Together, our study defines two functional states of free HDAC3, reveals the complete HDAC3 activation pathway, and links the C terminus function to the specific interaction between HDAC3 and DAD. These results also have implications in how signaling pathways may converge on the C terminus to regulate HDAC3 and suggest that the C terminus–mediated conformational change could represent a new target for inhibiting HDAC3 in diseases such as cancer.

In eukaryotic cells, histones are modified to regulate gene transcription (1). One such modification is acetylation of lysine residues of histones, which is controlled by histone acetyltransferases and histone deacetylases (HDACs) (2, 3, 4). Histone acetyltransferases acetylate lysine residues to activate transcription, whereas HDACs catalyze the reverse reaction, which is generally involved in transcriptional repression. The 18 mammalian HDACs can be divided into four classes. Class I HDACs (HDAC1, 2, 3, and 8) belong to the ubiquitously expressed, zinc-dependent deacetylases that are composed of a single HDAC domain and whose main function is to repress transcription. Class I HDACs are homologous to HDLP, a histone deacetylase–like protein expressed in a hyperthermophilic bacterium *Aquifex aeolicus* (5), suggesting that mammalian class I HDACs and the bacterial HDLP evolved from the same ancestor protein.

HDAC1, 2, and 3 are components of distinct multiprotein complexes (6). HDAC1 and HDAC2 are shared subunits of NURD, CoREST, Sin3, and other complexes, whereas HDAC3 is uniquely present in the multiprotein complex containing nuclear receptor corepressors (CoRs), including NCoR and silencing mediator of retinoic acid and thyroid hormone receptor (SMRT) (7, 8). These complexes also contain different SWI3, ADA2, NCoR or SMRT, and TFIIIB homology (SANT) domain proteins, which bind to and enhance the enzymatic activity of HDACs (9, 10, 11, 12). Free HDAC3 is thought to have minimal HDAC activity, whereas its interaction with the conserved SANT domain–containing deacetylase-activation domain (DAD) of NCoR/SMRT activates the latent enzymatic activity of HDAC3 (13, 14, 15, 16). Binding of HDAC3 to CoRs also confers HDAC3 with the ability to regulate signal-dependent transcription (17). Dysregulation of the HDAC3-dependent gene transcription is associated with various diseases such as cancer (18).

The HDAC3–DAD complex also contains inositol tetraphosphate (IP4) acting as a regulatory and structural component (11). HDAC3 simultaneously binds to DAD and IP4 in part through its N-terminal residues (amino acids 9–49), which form H1, H2, L1, and S2 structures (H, helix; L, loop; S, strand). Mutating Lys 25 in L1, which specifically binds to IP4, diminished DAD interaction and the deacetylase activity of HDAC3, demonstrating the important intermolecular glue function of IP4 (11). It has been proposed that IP4 and DAD interactions with HDAC3 allosterically increase substrate accessibility of HDAC3, resulting in HDAC3 activation (11, 19, 20). The active site of HDAC3 has a tunnel-like structure formed by loops L1–L7, as seen in HDLP and other class I HDACs (11, 19, 21, 22, 23, 24).

HDAC3 contains a unique C-terminal region not conserved in other HDACs (Fig. S1). Previous studies have provided evidence that this C-terminal region contributes to the ability of HDAC3 to bind to DAD and to deacetylate histones (13, 25, 26). However, the underlying mechanisms are poorly understood. The C-terminal region of HDAC3 is missing in the 3D structure of the HDAC3–DAD–IP4 complex owing to proteolysis after complex formation (11). This does not affect the conformation and activity of the HDAC3 complex (11). Based on these results, we hypothesized that the HDAC3 C terminus has a context-dependent function. Supporting this idea, we show here that the C terminus is required for HDAC3 activation before but not after a C terminus–dependent conformational change. C-terminal mutations prevent this conformational change to prevent HDAC3 activation. Mechanistically, we demonstrate that a C-terminus function is required for proper complex formation of HDAC3 with DAD and IP4 by allowing HDAC3 to undergo IP4-dependent interaction with DAD. This study clarifies the role of the HDAC3 C terminus, reveals the complete HDAC3 activation pathway, and surprisingly shows that the function of the unique C terminus is linked to the specific interaction between HDAC3 and DAD.

## Results

### *De novo* C-terminal truncations abolish HDAC3 activation by DAD and IP4

**Figure 1:**
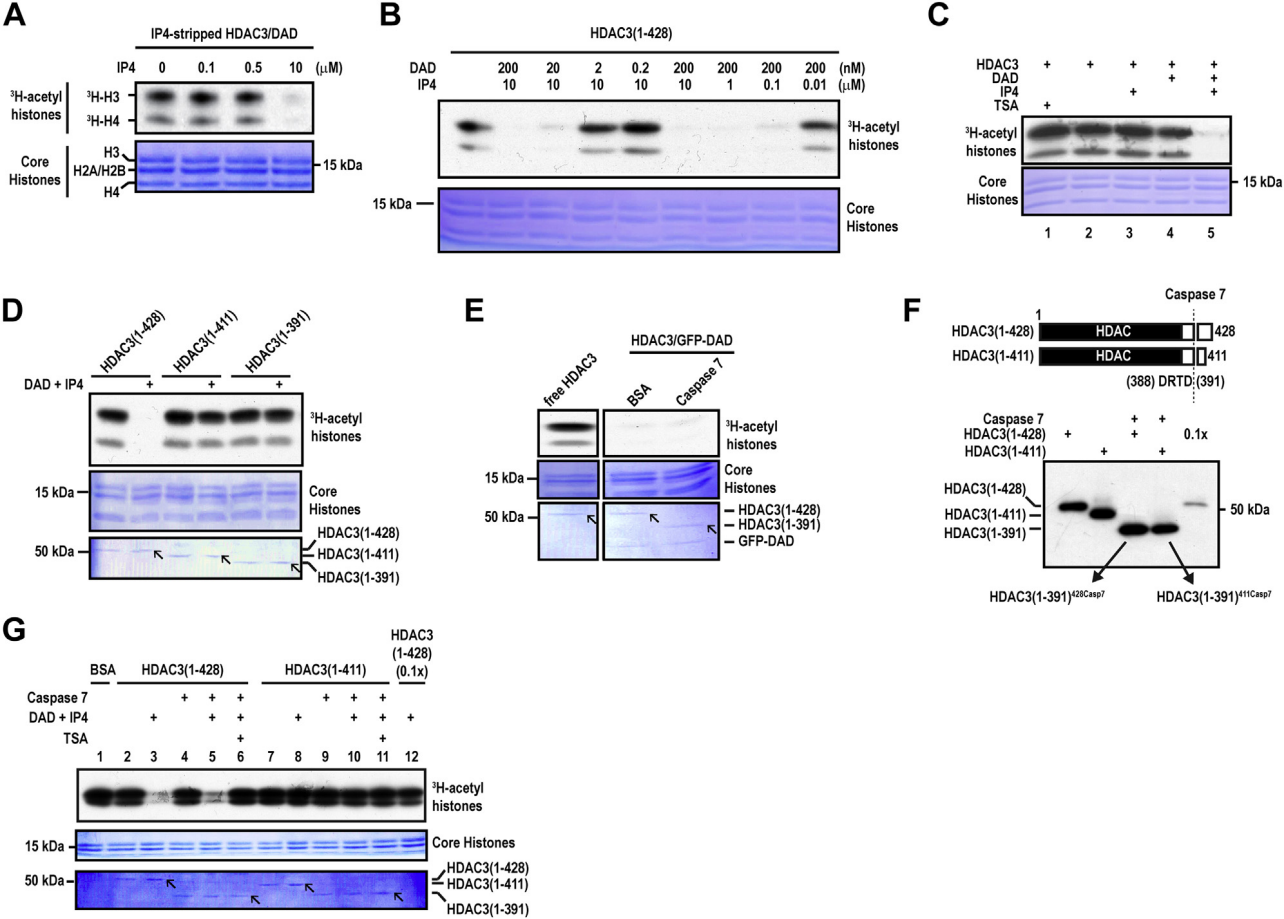
***De novo* C-terminal truncations abolish HDAC3 activation by DAD and IP4**. *A–C*, autoradiographic HDAC assays showing that both DAD and IP4 are required for HDAC3 activation. *D*, autoradiographic HDAC assays showing that recombinant HDAC3 lacking the C terminus cannot be activated by DAD and IP4. *E*, autoradiographic HDAC assays show that removing the HDAC3 C-terminal region after complex formation does not affect the activity of a preassembled HDAC3–DAD complex. *F*, western blot analyses of Caspase 7-cleaved HDAC3(1–428) and HDAC3(1–411), using the anti-HDAC3-N N19 antibody. The *bottom panel* shows Western blot of the indicated proteins using the N19 antibody. *G*, autoradiographic HDAC assays show that posttranslational removal of the C-terminal region does not affect the ability of free HDAC3 to be activated by DAD and IP4. In lanes 4 to 6 and 9 to 11, HDAC3(1–428) and HDAC3(1–411) had been pretreated with Caspase-7 to cleave the C-terminal tail. The C terminus was then depleted by anti-FLAG M2-agarose prior to the HDAC assay shown in the figure. In all figures except Figure 1*F*, the *bottom panels* show Coomassie Blue staining of core histones and HDAC3 proteins. In *D*, *E*, and *G*, the location of the wildtype and mutant HDAC3 protein bands were marked by *arrows*. DAD, deacetylase-activation domain; HDAC, histone deacetylase; IP4, inositol tetraphosphate; TSA, trichostatin A.

A reconstituted assay using recombinant proteins purified from baculovirus-infected insect cells (Fig. S2) was set up to facilitate the structure–function studies of HDAC3. We first asked if IP4 is important for the deacetylase activity of the HDAC3–DAD complex, which is known to be enzymatically active (13, 25). Stripping IP4 using a high-salt exchange method (9) reduced the activity of the complex (Fig. S3, lanes 2 *versus* 4), and this reduced activity was restored by exogenous IP4 (Figs. 1*A* and S3). In Figure 1*B*, recombinant free HDAC3 was dose dependently activated by DAD and IP4. In Figure 1*C*, Trichostatin A (TSA), an HDAC-specific inhibitor, was used to mark the total acetyl-histone substrate level (lane 1), which allowed us to show that all three components, HDAC3, DAD, and IP4, were required to reconstitute a robust HDAC activity (lane 5).

Using this assay, we compared FL (full-length) HDAC3(1–428) and its C-terminally truncated derivatives, including HDAC3(1–411) and HDAC3(1–391). Unlike the FL protein, HDAC3(1–411) and HDAC3(1–391) cannot be activated by DAD and IP4 (Fig. 1*D*). HDAC3(1–370) harboring a larger C-terminal truncation showed a similar result (data not shown).

### Posttranslational removal of the C terminus from FL HDAC3 does not affect HDAC3 activation

It has been reported that caspase 7 cleaves HDAC3 at D391 *in vivo* and *in vitro* (27, 28). We thus used caspase 7 to cleave the C-terminal region (amino acids 392–428) from both DAD-bound HDAC3 (Fig. 1*E*) and recombinant free HDAC3 (Fig. S4).

Cleaving the C terminus from DAD-bound HDAC3 had no effect on the HDAC activity of the complex (Fig. 1*E*), consistent with the previous observation that proteolytic cleavage of the C-terminal region also did not affect the activity of the HDAC3–DAD–IP4 complex (11).

Cleaving the C terminus from HDAC3(1–428) and HDAC3(1–411) both generated HDAC3(1–391) with the same primary structure, designated as HDAC3(1–391)^428Casp7^ and HDAC3(1–391)^411Casp7^, respectively (Fig. 1*F*). The C-terminal tail was cleared from the reaction *via* anti-FLAG M2-agarose depletion (Fig. S5).

Removing the C terminus from HDAC3(1–428) also had no effect on its ability to be activated by DAD and IP4 (Fig. 1*G*, lanes 2–5). FL HDAC3 and HDAC3(1–391)^428Casp7^ were similarly activated by DAD and IP4, showing a deacetylase activity far greater than that of FL HDAC3 used at a reduced dose (lane 12). Furthermore, TSA abolished the activity of HDAC3(1–391)^428Casp7^ (lane 6). Neither HDAC3(1–411) nor HDAC3(1–391)^411Casp7^ was activated by DAD and IP4 (lanes 7–10), and TSA had no effects on the activity of HDAC3(1–391)^411Casp7^ (lane 11).

A plausible explanation for these results is that a C terminus–dependent conformational change is required for DAD/IP4–mediated activation of HDAC3. HDAC3(1–411) and HDAC3(1–391)^411Casp7^ have an inactive conformation, which cannot be activated by DAD and IP4. The results also indicate that the conformation of FL HDAC3 is stable and can tolerate the loss of the C terminus for HDAC3 activation, supporting the idea that the C terminus function is conformation dependent.

### FL HDAC3 has a partially active conformation

As the first step to explore the role of the C terminus in regulating the conformation of HDAC3, we examined the conformational changes of HDAC3 associated with activation by comparing free FL HDAC3 and the HDAC3–DAD complex using partial trypsin digestion assays.

**Figure 2:**
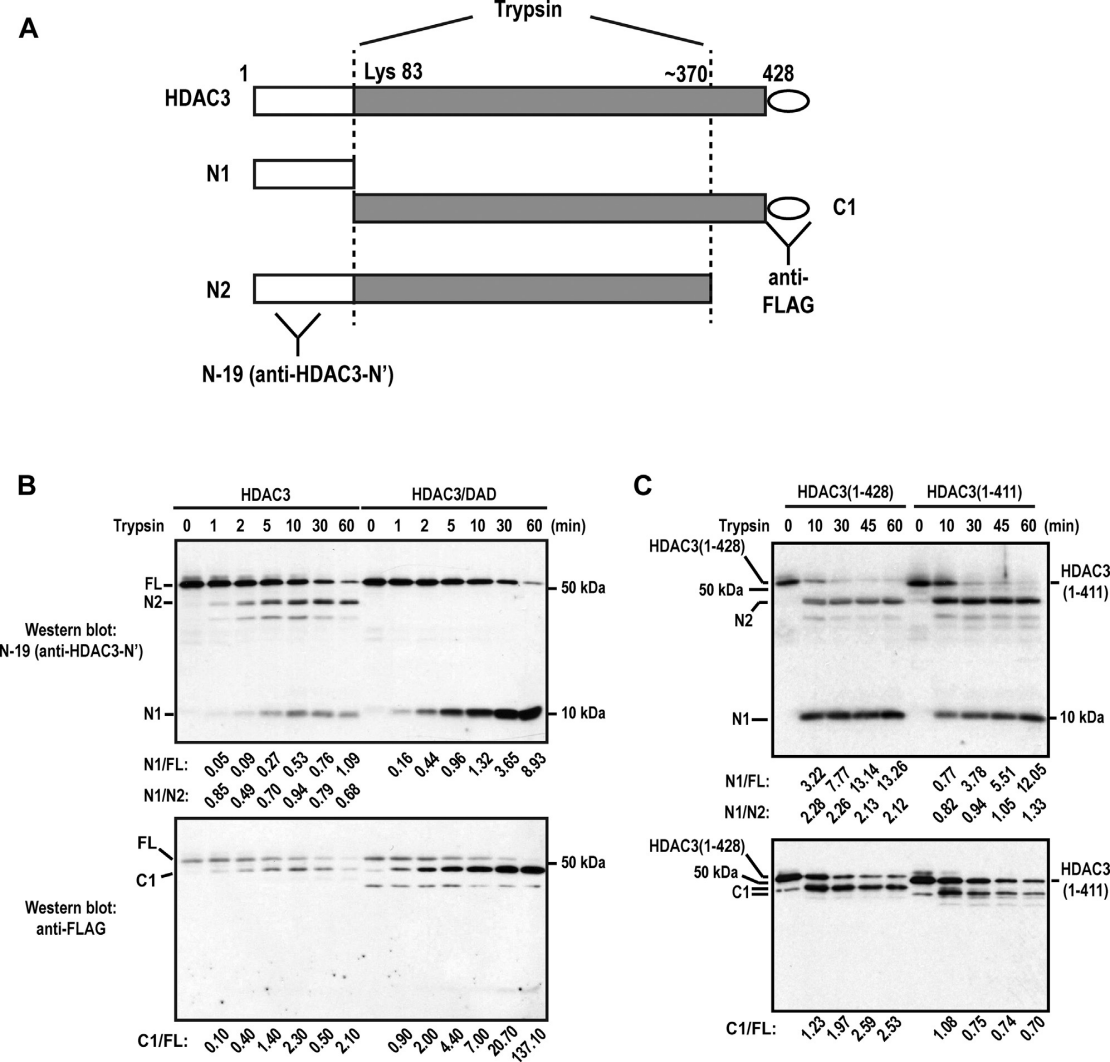
**Partial trypsin digestion analyses of free HDAC3(1–428), DAD-bound HDAC3(1–428) and free HDAC3(1-411).***A*, a diagram showing that trypsin digests HDAC3 at N- and C-terminal sites. The generated N-terminus-containing fragments (N1 and N2) were detected by the anti-HDAC3 N-terminus antibody (N-19). A C-terminus-containing fragment (C1) was detected by the anti-FLAG antibody *via* the C-terminus-fused FLAG tag. *B*, trypsin digestion of free HDAC3 and HDAC3/DAD complex. *C*, trypsin cleavage of free HDAC3(1–428) and HDAC3(1–411). The *bottom* shows NIH-Image J-quantified ratios of N1 *versus* N2, N1 *versus* the remaining uncut sample, or C1 *versus* the remaining uncut sample.

Trypsin cleaved an N-terminal residue (which we determined to be Lys 83, see below Fig. S6*A*) and a C-terminal residue near amino acid 370 (Fig. 2*A*). These cleavages generated N1 (∼10 kDa), C1 (∼40 kDa), and N2 (∼37 kDa) fragments, which were detected by using an anti-HDAC3-N′ antibody (N-19) (N1, N2) and an anti-FLAG antibody (C1) (Fig. 2*A*).

Compared with free HDAC3, the HDAC3–DAD complex produced higher levels of N1 and C1 (Fig. 2*B*), suggesting that active HDAC3 is more susceptible to trypsin digestion at the N-terminal site. Consistently, the ratios of N1/FL (uncut sample) and C1/FL were higher for HDAC3–DAD compared with free HDAC3. N2 was absent in digested HDAC3–DAD, indicating that complex formation protected the C-terminal site from trypsin digestion. By mutating Lys 83 to Ala, we mapped the N-terminal cleavage site to Lys 83 (Fig. S6*A*).

Compared with free HDAC3(1–411), free FL HDAC3 also produced higher levels of N1 and C1 and lower levels of N2 (Fig. 2*C*). Consistently, the ratios of N1/FL, C1/FL, and N1/N2 were higher for FL HDAC3. This shows that, compared with HDAC3(1–411), FL HDAC3 was also more susceptible to trypsin cleavage at the N-terminal site and resistant to trypsin cleavage at the C-terminal site. Preferential cleavage at the N-terminal site was also observed in FL HDAC3 compared with HDAC3(1–391) or HDAC3(1–370) (Fig. S6*B*). A similar result was also obtained comparing FL HDAC3 with an HDAC3 C-terminal point mutant (see below). We found that partial trypsin digestion assays were sensitive to assay conditions. As a result, cleavage efficiencies may vary between different experiments. However, the results were comparable between different samples in the same experiment.

**Figure 3:**
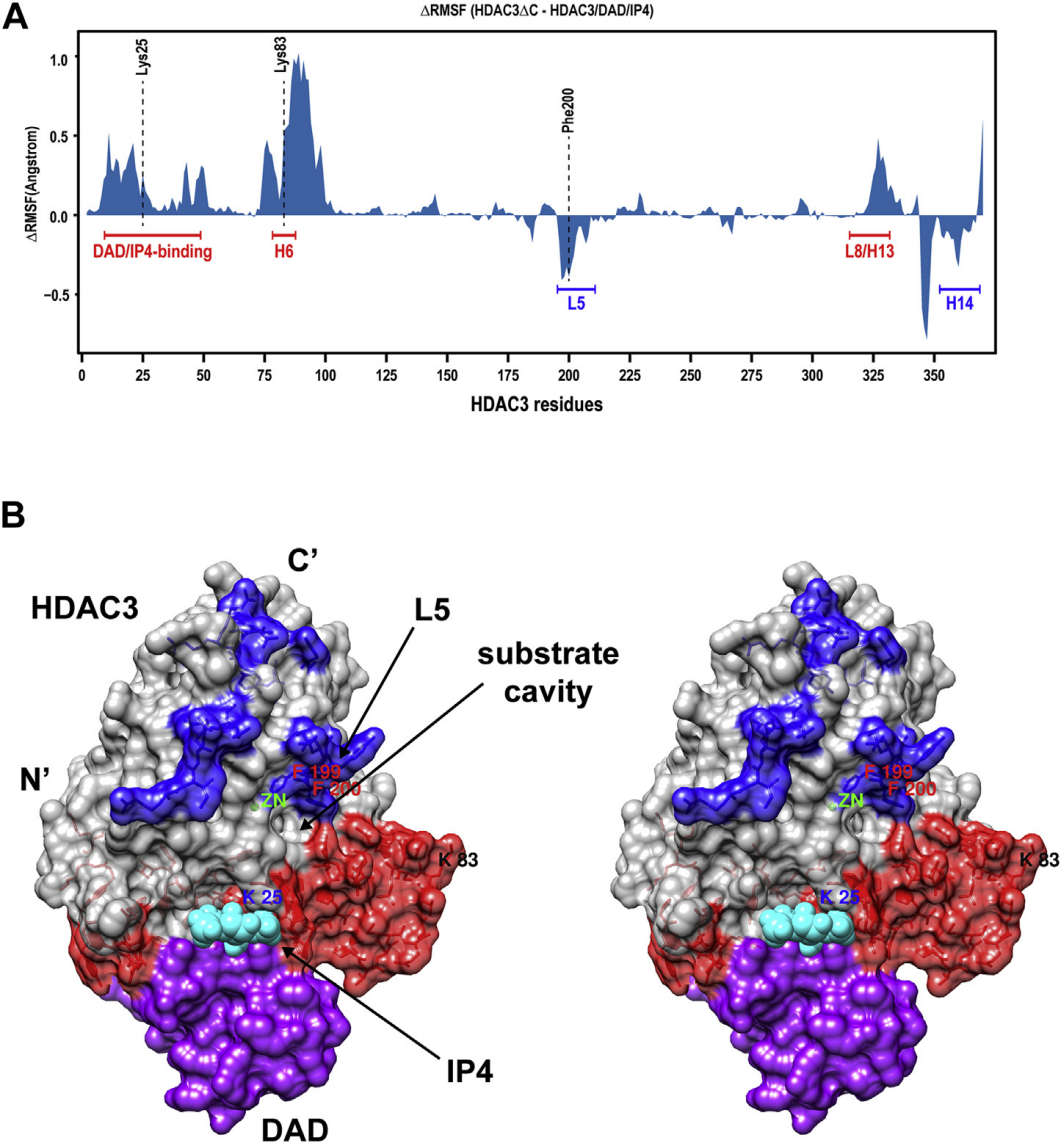
**Active conformational changes affect DAD/IP4 binding surfaces and the active site.***A*, relative RMSFs from AMBER molecular dynamics studies between HDAC3ΔC and the HDAC3/DAD/IP4 complex. A positive value denotes unstable conformation in HDAC3ΔC and a negative value denotes unstable conformation in the DAD/IP4-bound HDAC3. *B*, wall-eye (or relaxed) stereo images of HDAC3/DAD/IP4 complex highlighting conformational differences between HDAC3ΔC and the DAD/IP4–bound HDAC3. Amino acids with greater than 0.145 Å differences in ΔRMSF (except the extreme C-terminal amino acid 369–370) were colored in red for a positive ΔRMSF or blue for a negative ΔRMSF. The location of DAD, IP4, substrate cavity, zinc, L5, and residues K25, K83, F199, and F200 and the orientations of N and C termini were also shown. The cutoff 0.145 Å was chosen based on the ΔRMSF plot (*A*) to color only the major peak residues (including those in the N-terminal and active-site regions). DAD, deacetylase-activation domain; HDAC, histone deacetylase; IP4, inositol tetraphosphate; RMSF, root-mean-square fluctuation.

**Figure 4:**
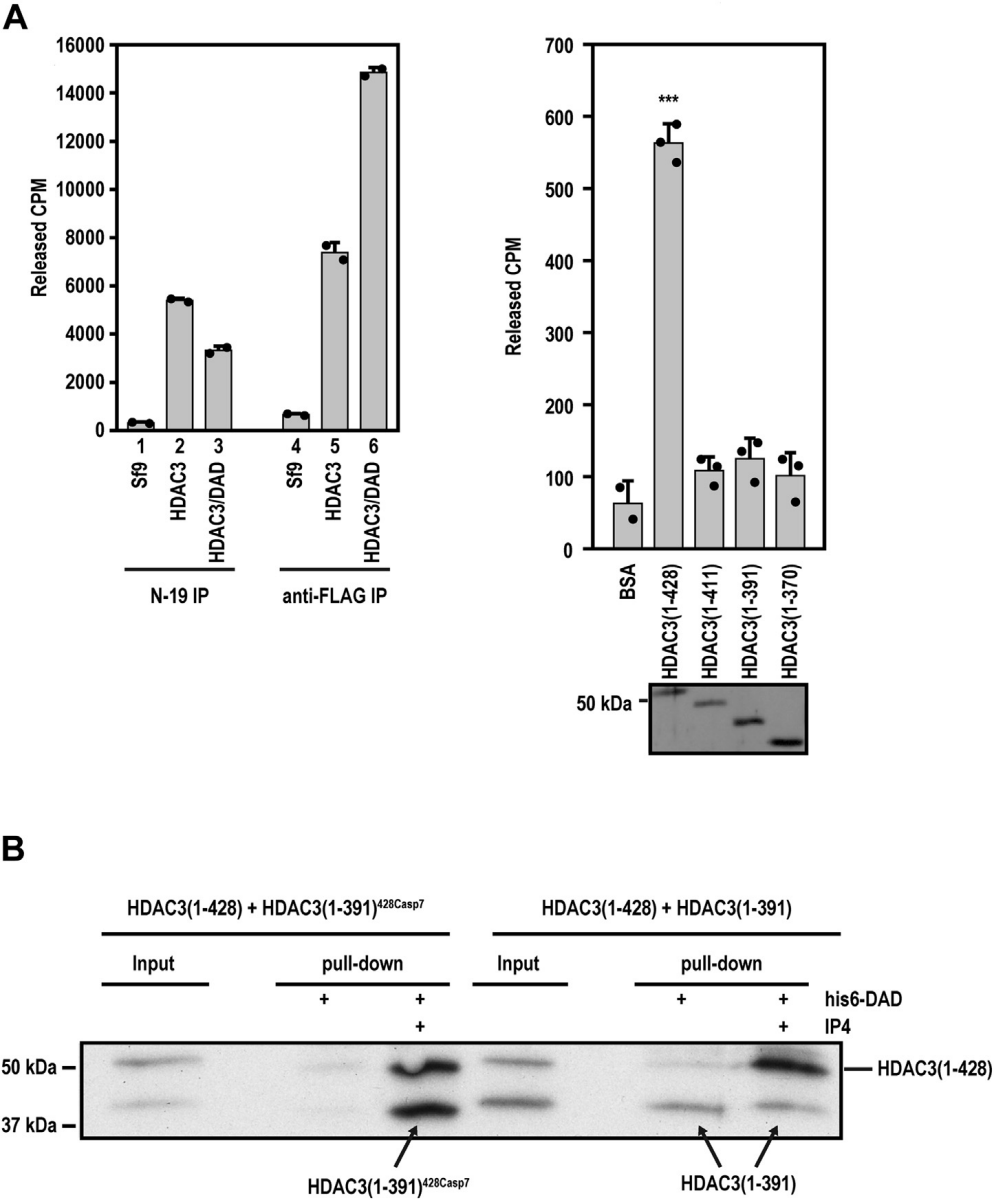
**Loss of the C terminus abolishes the basal HDAC activity of free HDAC3 and its IP4-dependent interaction with DAD.***A*, *left*, liquid-based HDAC assays of N-19 and anti-FLAG immunoprecipitates derived from Sf9 cells infected with baculovirus expressing HDAC3 alone or both HDAC3 and DAD (n = 2). Uninfected cells are used as the control. The results show mean + standard deviation. *Right*, liquid-based HDAC assays of FL and C-terminally truncated HDAC3 as free proteins. The results show mean + standard deviation (n = 2 for BSA, n = 3 for other samples). ∗∗∗*p* ≤ 0.001 between HDAC3(1–428) and each of the other samples (two-tailed Student's *t* test). *B*, pull-down of the indicated HDAC3 proteins by His-tagged DAD in the absence or presence of IP4. HDAC3(1–428) is mixed with its caspase 7-cleaved product (*left*) or with recombinant HDAC3(1–391) (*right*). BSA, bovine serum albumin; DAD, deacetylase-activation domain; HDAC, histone deacetylase; IP4, inositol tetraphosphate.

**Figure 5:**
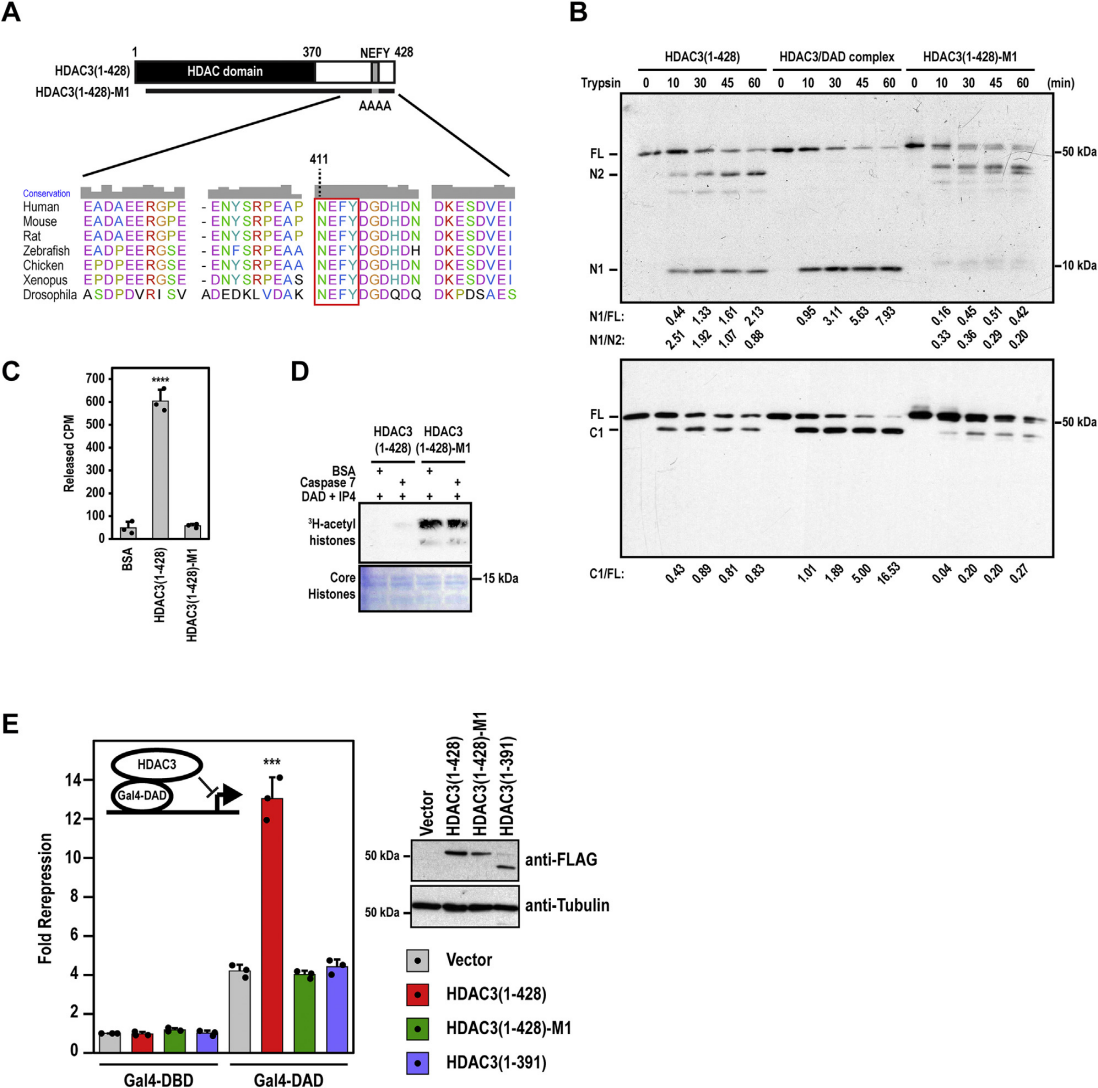
**Sequence-dependent C terminus function and its requirement for DAD activation of HDAC3 *in vivo*.***A*, a diagram showing the location of NEFY(411–414), which are mutated to AAAA in HDAC3-M1. *Bottom*, NEFY(411–414) is conserved across species. *B*, analysis of the conformational state of HDAC3(1–428)-M1 by partial trypsin digestion assay, along with free WT HDAC3 (*left*) and the HDAC3–DAD complex (*middle*). *C* and *D*, HDAC activity assays of HDAC3(1–428)-M1 along with the wildtype HDAC3 using liquid scintillation (*C*) and autoradiographic (*D*) HDAC assays. In *C*, the results show mean + standard deviation (n = 3). ∗∗∗∗*p* ≤ 0.0001 between HDAC3(1–428) and each of the other two samples (two-tailed Student's *t* test). *E*, HEK293T cells are transfected with a Gal4-dependent luciferase reporter along with expression plasmids for Gal4–DBD, Gal4–DAD, wildtype or mutant HDAC3 (n = 3). The *y*-axis denotes fold repression of luciferase activities (Gal4–DBD/Gal4–DAD). The results show mean + standard deviation. ∗∗∗*p* ≤ 0.001 between HDAC3(1–428) and each of the other samples expressing Gal4–DAD (two-tailed Student's *t* test). DAD, deacetylase-activation domain; DBD, DNA-binding domain; HDAC, histone deacetylase; IP4, inositol tetraphosphate.

Together, these results show that free FL HDAC3 indeed has a different conformation from that of the C-terminal mutants, supporting the hypothesis that the HDAC3 C terminus regulates HDAC3 conformation to regulate HDAC3 activation. The results also show that the C terminus can drive a conformational change similar to that associated with fully activated HDAC3, albeit to a lesser extent.

### HDAC3 conformational changes affect DAD/IP4–binding surfaces and the active site

Since DAD/IP4–mediated activation and HDAC3 C terminus produced similar conformational changes of HDAC3, we next sought to locate the sites of these changes by performing molecular dynamics simulation studies. Specifically, we compared HDAC3 alone (referred to as HDAC3ΔC) and the entire HDAC3–DAD–IP4 complex using the coordinates of the complex (Protein Data Bank# 4A69) (11). Given that HDAC3 in this structure lacks the C-terminal region (377–428), the simulation of HDAC3ΔC should reflect the conformational stability of the truncated HDAC3 that cannot be activated by DAD and IP4. On the other hand, because removing the C-terminal region of HDAC3 does not affect the active conformation of crystalized HDAC3–DAD–IP4 complex, the simulation result of this complex should reflect the conformational stability of the active HDAC3.

Figure 3*A* shows the relative root-mean-square fluctuations per residue between HDACΔC and HDAC3–DAD–IP4. The N-terminal region of HDAC3ΔC was highly unstable compared with HDAC3–DAD–IP4. Of note, this included helix H6 (amino acids 78–88) surrounding the trypsin cleavage site Lys 83, which explains why inactive HDAC3 was resistant to trypsin cleavage at Lys 83. Unstable conformation was also observed in the DAD/IP4–binding surface (amino acids 9–49), including Lys 25 (11). Intriguingly, most unstable regions in HDAC3ΔC were near DAD or IP4 and located at the same side of the substrate pocket as DAD and IP4 (Fig. 3*B*, in red, Movie S1).

The opposite side was enriched with residues with a more flexible conformation in the active state of HDAC3 (Fig. 3, *A* and *B*, colored in blue). This included loop L5 (amino acids 195–211), which is near zinc and forms part of the wall of the substrate pocket. The homologous residue of Phe 200 in HDLP marks the narrowest portion of the substrate tunnel and directly contacted TSA (21). When HDAC3 was aligned to TSA/HDLP, we found that Phe 200 was also within contact distance of TSA (3.917 Å from C15 and 4.023 Å from C14, Fig. S7). Our finding is consistent with the induced fit model (29) and suggests that certain active site residues have a flexible conformation in the active state of HDAC3. Lacking this flexible conformation may render HDAC3 inactive.

Together, these results reveal that the active HDAC3 conformational changes affect DAD/IP4–binding surfaces and the active site. Our finding that the N-terminal region is unstable is consistent with the results from a similar study reported previously (19). In addition, given that the active site is relatively far from DAD and IP4, its observed conformational changes are likely allosterically induced by the conformational changes at the DAD and IP4-binding sites.

### Evidence that free FL HDAC3 conformation is fundamentally different from that of the activation-defective C-terminal mutants

Although these conformational studies revealed measurable changes of HDAC3 conformation, we have shown that FL and the C-terminally truncated HDAC3 proteins have an all-or-none ability to be activated by DAD and IP4, promoting us to further explore their functional differences.

Given our trypsin digestion results showing that FL HDAC3 has an intermediate conformation between the completely inactive and fully active HDAC3, we wondered if it has a partial deacetylase activity. This was tested by using a sensitive liquid scintillation–based HDAC assay. Because the N-19 antibody only recognizes the free form of HDAC3 (25), we used N-19 to immunoprecipitate free HDAC3 from Sf9 cells infected with HDAC3 baculovirus alone or infected with both HDAC3 and DAD baculoviruses. Of note, N-19 immunoprecipitated a higher deacetylase activity from Sf9 cells expressing HDAC3 alone (Fig. 4*A*, left, lane 2 *versus* 3). In contrast, the anti-FLAG antibody, which recognized total HDAC3, immunoprecipitated a higher deacetylase activity from Sf9 cells expressing both HDAC3 and DAD (lane 5 *versus* 6). Given that most HDAC3 in the HDAC3/DAD–coexpressing cells should bind to DAD, these results further confirmed that N-19 recognized free HDAC3 and demonstrated that free HDAC3 is indeed active as a deacetylase. Michaelis–Menten analysis further showed that free FL HDAC3 followed the typical Michaelis–Menten kinetics (Fig. S8). TSA dramatically reduced the HDAC3 activity. Kinetic analysis of the TSA inhibition data indicates that it is also a competitive inhibitor for free HDAC3 (Fig. S8). This shows that the canonical substrate-binding pocket is indeed open in free HDAC3, which was occupied by TSA.

Next, comparing free FL HDAC3 and its C-terminal mutants showed that HDAC3(1–411), HDAC3(1–391), and HDAC3(1–370) all lacked significant basal activities (Fig. 4*A*, right). These functional assays thus show that the conformation of free FL HDAC3 is fundamentally different from that of the C-terminal mutants, indicating that all mutants are unable to pass a critical step in the conformational change of HDAC3 that is required for its deacetylase activity.

### The C terminus is required for IP4-dependent HDAC3 interaction with DAD

Given that the N-terminal region is important for IP4-dependent HDAC3 interaction with DAD (11), to gain further insight into the mechanism by which the C terminus regulates HDAC3 activation, we examined IP4-dependent HDAC3 interaction with DAD. Specifically, given that recombinant HDAC3(1–391), but not caspase 7-generated HDAC3(1–391), failed to be activated by DAD and IP4, we compared recombinant HDAC3(1–391) and HDAC3(1–391)^428Casp7^ for their abilities to undergo IP4-dependent DAD interaction, using free FL HDAC3(1–428) as a control.

HDAC3(1–428) and HDAC3(1–391)^428Casp7^, but not the recombinant HDAC3(1–391), were capable of mediating a strong IP4-dependent interaction with DAD (Fig. 4*B*). The weak interaction between HDAC3(1–391) and DAD has been previously reported (25). We now show that this interaction was not affected by IP4, indicating that HDAC3(1–391) forms a nonfunctional complex with DAD, which explains why it cannot be activated by DAD and IP4. On the other hand, HDAC3(1–391)^428Casp7^ showed a potent IP4-dependent DAD interaction, explaining why it can be activated by DAD and IP4. Thus, the C terminus–dependent conformational change enables HDAC3 activation by enabling IP4-dependent interaction of HDAC3 with DAD.

### The C-terminal function is sequence dependent

Given the highly conserved HDAC3 C-terminal sequence, we hypothesized that its function should depend on specific amino acid sequence. Aligning the different HDAC3 proteins revealed a highly conserved region that includes NEFY(411–414). We reasoned that NEFY may facilitate the C terminus–dependent conformational change by mediating various hydrophobic, hydrogen bond, or charged interactions. We therefore tested the above hypothesis by mutating NEFY(411–414) to Ala (Fig. 5*A*). Characterization of the generated HDAC3(1–428)-M1 mutant showed that it indeed had the inactive conformation. Partial trypsin digestion assays showed that HDAC3(1–428)-M1 was resistant to trypsin cleavage at the N-terminal site (Fig. 5*B*). Free WT (wildtype) HDAC3 clearly showed an intermediate conformation (Fig. 5*B*), providing further support of its partial deacetylase activity. Additional analyses showed that HDAC3(1–428)-M1 lacked a basal HDAC activity (Fig. 5*C*) and cannot be activated by DAD and IP4 (Fig. 5*D*).

### The C terminus is required for HDAC3 activation by DAD *in vivo*

To validate the results *in vivo*, we used a cell-based assay, which is capable of recapitulating DAD–HDAC3 complex-dependent repression of luciferase gene transcription *in vivo* (25). Gal4–DAD alone showed a modest repression activity (Fig. 5*E*), consistent with its interaction with endogenous HDAC3 (25). The activity of Gal4–DAD was significantly enhanced by ectopic FL HDAC3(1–428) but not HDAC3(1–428)-M1 or HDAC3(1–391) (Fig. 5*E*). Both HDAC3(1–428)-M1 and HDAC3(1–391) were expressed in transfected HEK293T cells at a level greater than that of endogenous HDAC3 (Fig. 5*E* and data not shown). The relatively lower expression levels of these C-terminal mutants compared with that of FL HDAC3 are consistent with a role of the C terminus in stabilizing HDAC3 protein expression (25). Together, these results are consistent with the conclusion that the C terminus is also required for DAD activation of HDAC3 *in vivo*.

## Discussion

**Figure 6:**
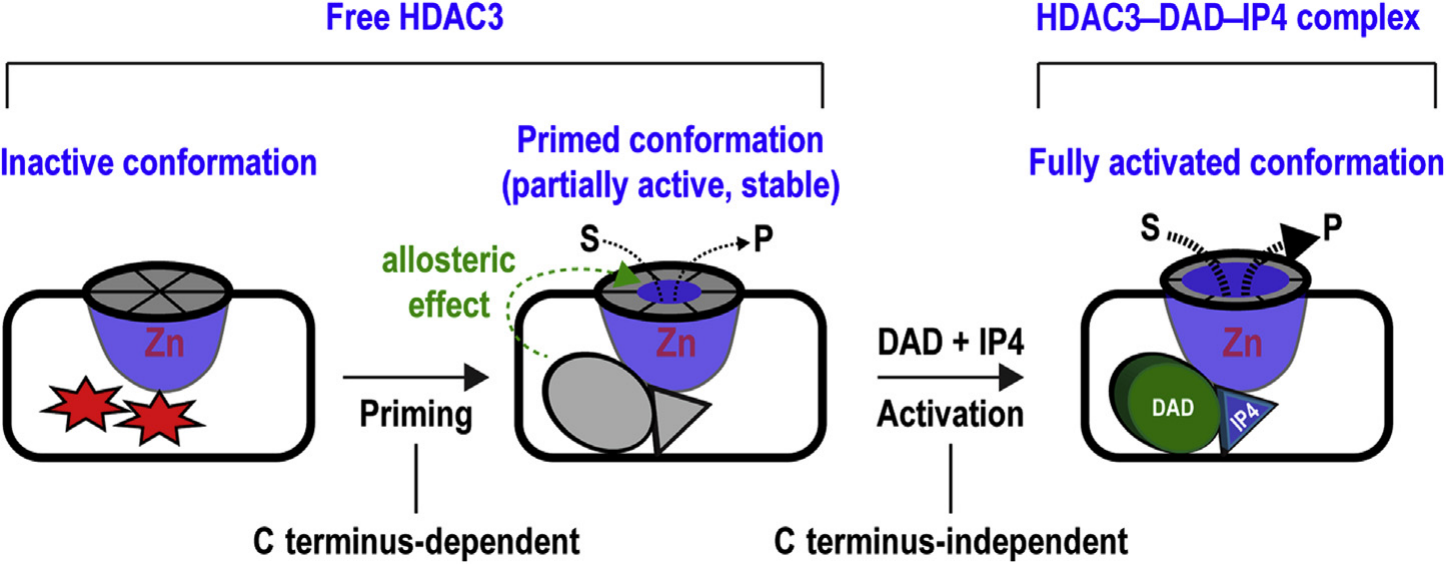
**A proposed model of the complete two-step HDAC3 activation pathway showing the different conformational states and cross talk between HDAC3 C terminus and DAD/IP4.** HDAC3 activation requires two distinct steps that variably depend on the C terminus. First, the C terminus primes HDAC3 by stabilizing the DAD/IP4–binding sites and by allosterically enhancing the substrate accessibility, making HDAC3 partially active and poised for activation by DAD and IP4. Second, the primed HDAC3 is activated by DAD and IP4 in a C terminus–independent manner. In the *left panel*, the *red-colored stars* represent unstable conformations of the DAD and IP4-binding sites. These conformations were stabilized after priming, making them ready for binding by DAD and IP4. This is shown by the *gray-colored ellipse* and *triangle* in the *middle panel* that match the shapes of DAD and IP4, respectively, shown on the *right*. For simplicity, the C terminus was not shown in these representations. See text for details. DAD, deacetylase-activation domain; HDAC, histone deacetylase; IP4, inositol tetraphosphate.

This study revealed novel functions of the C terminus of HDAC3 by showing that a previously unrecognized priming step mediated by the C terminus–dependent conformational change is required for DAD and IP4-mediated activation of HDAC3 (Fig. 6). We show that free HDAC3 has two conformational states. The inactive state cannot be directly activated by DAD and IP4. Remodeling HDAC3 conformation by the C terminus allows productive interaction between HDAC3 and DAD/IP4, thus revealing the complete HDAC3 activation pathway (Fig. 6). Our results also show that the conformational change is blocked by the C-terminal mutations, explaining why these mutations prevent HDAC3 activation. The C terminus, however, is dispensable after the priming step, indicating that the HDAC3 conformation is stable after priming. This also shows that DAD and IP4 can sense the conformational state of HDAC3, which may be important for preventing premature activation of HDAC3.

Intriguingly, both the HDAC3 C terminus and the DAD-mediated interaction are unique to HDAC3. Our results thus link the unique C terminus function of HDAC3 to its specific interaction with DAD. HDAC3 may have evolved the specific C-terminal region to allow its activation by DAD. This explains why HDLP and HDAC8 lack both a unique C-terminal region and an activation mechanism similar to that conferred by DAD. The different C-terminal regions of HDAC3 *versus* HDAC1/2 may also specify which SANT-domain proteins activate these HDACs. The HDAC3 C terminus only partially activates HDAC3. These observations suggest that both the C terminus– and the DAD-dependent steps and their cross talk are important for HDAC3 during evolution, which is also consistent with the highly conserved C terminus and DAD sequences across species (Fig. 5*A*) (13, 14).

It has been suggested that free HDAC3 adopts different conformations, most of which are inactive but shift toward the active conformation upon binding to DAD and IP4 (19). We show that removing the C terminus from free HDAC3 had no effects on its ability to be activated by DAD and IP4, suggesting that most (if not all) HDAC3 have the primed conformation and are poised for activation.

It has been shown that chaperone-mediated folding of HDAC3 is also required for DAD-mediated activation (15). The present study shows that HDAC3 activation also requires the C terminus–dependent conformational change. The relationship between folding and this C terminus function remains to be explored in future studies. The chaperone-mediated folding of HDAC3 is required for DAD interaction (15). We have shown that the inactive HDAC3 lacking the C terminus can still bind to DAD (Fig. 4*B*). Furthermore, the mammalian HDAC8 lacks a specific C-terminal region but is enzymatically active, suggesting that a unique C-terminal region is dispensable for the folding of the conserved HDAC domain. Therefore, we favor the idea that the HDAC3 C terminus functions at a step after folding to regulate HDAC3 conformation and DAD interaction. This may allow the C terminus to play a gatekeeper role in controlling HDAC3 activation, in a manner analogous to the C terminus of nuclear receptors, whose conformational change is also required for nuclear receptors activation by regulating coregulator interactions (30). A regulatory role of the C terminus is also supported by the finding that the C terminus is posttranslationally modified to regulate HDAC3 function (see below).

Our results indicate that the function of the C terminus in HDAC3 activation is attributed to its ability to regulate HDAC3 conformation. Although these studies have mainly characterized the HDAC3 proteins expressed in Sf9 cells, multiple results support a similar function of the HDAC3 C terminus in human cells. Recombinant HDAC3 C-terminal mutants cannot be activated by DAD in HEK293T cells (Fig. 5*E*). *De novo* C-terminal truncations also diminished the deacetylase activity of the transfected HDAC3 mutant proteins in HeLa cells (26). In contrast, losing the entire C terminus from a preassembled HDAC3–DAD–IP4 complex in HEK293T cells had no effect on the deacetylase activity of HDAC3 or its ability to bind to DAD and IP4 (11). Our results also show that the C terminus regulates HDAC3 and DAD interaction. Consistent with a similar function of the C terminus in human cells, we have previously shown that deleting the C-terminal region of HDAC3 resulted in a misassembled HDAC3–DAD complex in HEK293T cells that was both unstable and enzymatically inactive (25), consistent with our finding that the C terminus is required for IP4-dependent HDAC3 and DAD interaction (Fig. 4*B*).

How might the C terminus regulate HDAC3 conformation? Given our finding that the C terminus stabilizes the N-terminal conformation, one possibility is that the C terminus is involved in the N-C interaction, as often found in various other proteins (31, 32, 33). Supporting this idea, we have shown that NEFY(411–414) is required for the C terminus–dependent conformational change. Conceivably, NEFY(411–414), possibly in conjunction with other C-terminal sequences, may fold back and contact the N-terminal and/or other regions to stabilize their conformation, allowing the cooperative binding and activation of HDAC3 by DAD and IP4. The fact that NEFY(411–414) is missing in all assayed C-terminal mutants explains why none of these mutants can be activated by DAD and IP4.

Conformational changes at the DAD-IP4–binding sites may also allosterically affect the active site conformation, leading to increased substrate accessibility. This explains why free FL HDAC3, but not the C-terminal mutants, is enzymatically active. An allosteric mechanism may also explain why the primed HDAC3 conformation can tolerate the subsequent loss of the C terminus. A partial deacetylase activity of free HDAC3 is surprising, given that HDAC3 is thought to strictly depend on interactions with DAD and IP4 for its enzymatic activity (13, 14, 15, 16). However, previous studies may not completely exclude the possibility that free HDAC3 is active. Many of these studies were focused on comparing free HDAC3 with the fully activated DAD–HDAC3–IP4 complex. In our studies, the failure to detect a significant deacetylase activity of free HDAC3 using the gel-based assay may be related to the lower sensitivity of this assay and its less quantitative nature compared with the liquid scintillation–based assay. Furthermore, other class I HDACs and HDLP are enzymatically active as a free protein (21, 34). Therefore, HDAC3 may not be fundamentally different from other class I HDACs regarding the activity of free proteins as previously thought.

Consistent with our molecular dynamics simulation results, a previous study (19) also observed the unstable conformations of free HDAC3 at the N-terminal region including Lys 25 and Lys 83. However, there are some differences between this study and ours. In addition to the N-terminal region, this study also found that L6 was unstable in HDAC3ΔC, albeit to a lesser degree compared with the N terminus. On the other hand, we found that L5 was unstable in the HDAC3–DAD–IP4 complex. Both L5 and L6 are in close proximity to the active site zinc. Therefore, these differences may be related to the use of different methods to simulate the zinc center. The previous study used a covalently bonded model, whereas ours used a nonbonded cationic dummy atom method (35). Using a nonbonded model may help reveal the unstable conformation caused by DAD and IP4 on the active site. An unstable conformation of L6 is still consistent with our model, suggesting that such a conformation, and in conjunction with that of the N-terminal region, may jointly contribute to the failure of the inactive HDAC3 to properly bind to DAD and IP4. On the other hand, our results are consistent with the induced fit idea and suggest that a relaxed conformation of certain active site residues is important for the deacetylase activity of HDAC3.

The HDAC3 C terminus–dependent conformational change may also serve as a target of upstream signaling pathways. Phosphorylation of Ser 424 in HDAC3 enhances its deacetylase activity, whereas dephosphorylation at this serine residue negatively regulates HDAC3 activity (36). Our study supports the idea that phosphorylation/dephosphorylation of Ser 424 regulates HDAC3 activity by regulating HDAC3 conformation (36). HDAC3 has also been shown to be cleaved at D391 under certain stress conditions (27, 28). However, there are conflicting reports about the consequence of this cleavage on HDAC3 functions (27, 28). These discrepancies may be explained by the HDAC3 conformation. If cleavage occurs prior to the conformational change, it should inactivate HDAC3. Contrarily, if cleavage occurs after the conformational change, and if the conformation is also stable *in vivo* as observed *in vitro*, it should not affect and may even increase the activity of HDAC3. Conceivably, depleting the C terminus may eliminate possible inhibitory effects conferred by unknown factors or modifications associated with the C terminus.

HDAC3 has been reported to have deacetylase-independent functions (37, 38, 39). The literature thus far has supported the idea that CoRs are required for both deacetylase-dependent and deacetylase-independent functions of HDAC3 (16, 38). Given that DAD is the main HDAC3-binding site of CoRs, along with our finding that the C terminus is required for the productive interaction between HDAC3 and DAD, targeting C terminus–mediated conformational change may be a viable strategy to inhibit the total function of HDAC3. Given that the HDAC3 C terminus is not conserved in other HDACs, it may be feasible to specifically inhibit the function of the HDAC3 C terminus. This may achieve selective inhibition of HDAC3, thus overcoming the limitation of current HDAC inhibitors.

## Experimental procedures

### Cell culture and luciferase assay

HEK293T cells were maintained in Dulbecco's modified Eagle's medium with 10% FBS. Luciferase reporter assays were performed in HEK293T cells seeded in 24-well plates. The cells were transfected with equal total amounts of plasmids using X-tremeGENE HP DNA Transfection Reagent (MilliporeSigma), and luciferase assays were performed 36 h later. The specific amounts of plasmids used for transfection are 100 ng HDAC3 and HDAC3 mutants, 100 ng Gal4-DBD (DNA-binding domain) and Gal4-DAD (deacetylase-activation domain from NCoR), and 25 ng Gal4-UASx5 luciferase reporter. Luciferase units (Promega) were normalized to β-gal (10 ng) activity, which served as an internal control for normalization of the transfection efficiency. Fold repression was relative to the luciferase activity observed with Gal4-DBD–transfected cells. Results are presented as mean + standard deviation from replicated samples in representative experiments.

### Chemicals and antibodies

Trichostatin A (TSA) and D-myo-inositol-1,4,5,6-tetraphosphate (IP4(1,4,5,6)) were obtained from Cayman. The anti-HDAC3-N′ N-19 antibody was from Santa Cruz Biotechnology. Anti-FLAG M2-agarose and anti-FLAG antibody were from MilliporeSigma.

### Protein expression

Recombinant proteins were expressed and purified from Sf9 insect cells following infection with pFastBac (Invitrogen) (25). The expression vectors have been described (25) or constructed following standard molecular cloning and PCR techniques. The mutations were confirmed by Sanger sequencing. The mouse NCoR-derived DAD domain (mNCoR 389–498) was purified *via* a C-terminal His tag. All other proteins were purified *via* an in-frame C-terminal FLAG tag using anti-FLAG M2-agarose beads (25).

### Histone deacetylation activity assays

*Xenopus* histone was acetylated with ^3^H-acetyl CoA (PerkinElmer) using a recombinant p300 as previously described (25). Unincorporated ^3^H-acetyl CoA was removed from p300-acetylated histones by dialysis using Slide-A-Lyzer Midi Dialysis Units (3500 MWCO, MilliporeSigma) before being used for histone deacetylation assay. Histone deacetylation assays were carried out at 30 °C for 60 min using 100 ng of WT (wildtype) or mutant HDAC3 proteins, 200 nM DAD, and 1 μM IP4 unless otherwise noted. For the gel-based assay, the product was detected by autoradiography following polyacrylamide gel electrophoresis (PAGE). For the liquid-based assay, ethyl acetate (MilliporeSigma) was used to extract the released ^3^H-acetic acid, which was quantified by liquid scintillation.

### Caspase 7 expression, cleavage, and trypsin digestion assays

Recombinant active caspase 7 was expressed from a pET23b-Casp7-His plasmid, kindly provided by Guy Salvesen (Addgene plasmid # 11825; http://n2t.net/addgene:11825; RRID: Addgene_11825). Caspase 7 cleavage assay was performed at 37 °C for 1 h. Limited trypsin digestion assay was performed at room temperature as described (40), and 400 ng of WT or mutant HDAC3 proteins were used. Trypsin was from ThermoFisher (Pierce Trypsin Protease).

### Molecular dynamics simulation

Molecular dynamics simulation was performed using the PMEMD module of the Amber 2018 simulation package (41). The coordinates of HDAC3–DAD–IP4 (4A69) complex were downloaded from Protein Data Bank (https://www.rcsb.org/) and used as the initial state of HDAC3ΔC and the HDAC3–DAD–IP4 complex. Amber ff99SBildn was used as the force field. Protonation states were assigned by the H++ server (42) and as described (19). A nonbonded cationic dummy atom method (35) was used to simulate the zinc center, including its coordinated His and a hydroxide that replaces the acetate bound to zinc in the 3D structure of the complex. IP4 was prepared using the Amber antechamber tool and subsequently optimized using Gaussian (g16) with the B3LYP/6-31g∗ basic set. Parameters of molecular dynamics simulation were similar to those described in the previous study (19). The system was first solvated in a 10.0 Å TIP3P box, and KCl was added to a 150 mM concentration. Energy minimization was performed, followed by eight 150-ps steps of heating under the constant volume (NVT) ensemble which increased the temperature from 0 to 300 K. The system was then equilibrated under the constant pressure (NPT) ensemble at the 300 K temperature for 100 ps. All production runs were performed using a GTX-1080Ti GPU under NVT at the 300 K temperature for 100 ns with a 2-fs time step. Trajectories were analyzed using the pytraj software (43) and further processed in R. Images were prepared using the UCSF Chimera software (44).

## Supporting information

This article contains supporting information.

## Conflict of interest

The authors declare that they have no conflicts of interest with the contents of this article.
