# Supplementary material for: A C terminus–dependent conformational change is required for HDAC3 activation by nuclear receptor corepressors
Source: J Biol Chem. 2021 Sep 11;297(4):101192. doi: 10.1016/j.jbc.2021.101192 (PMC8502911; doi:10.1016/j.jbc.2021.101192)
Supplement: Figures S1–S8 [file mmc2.pdf]

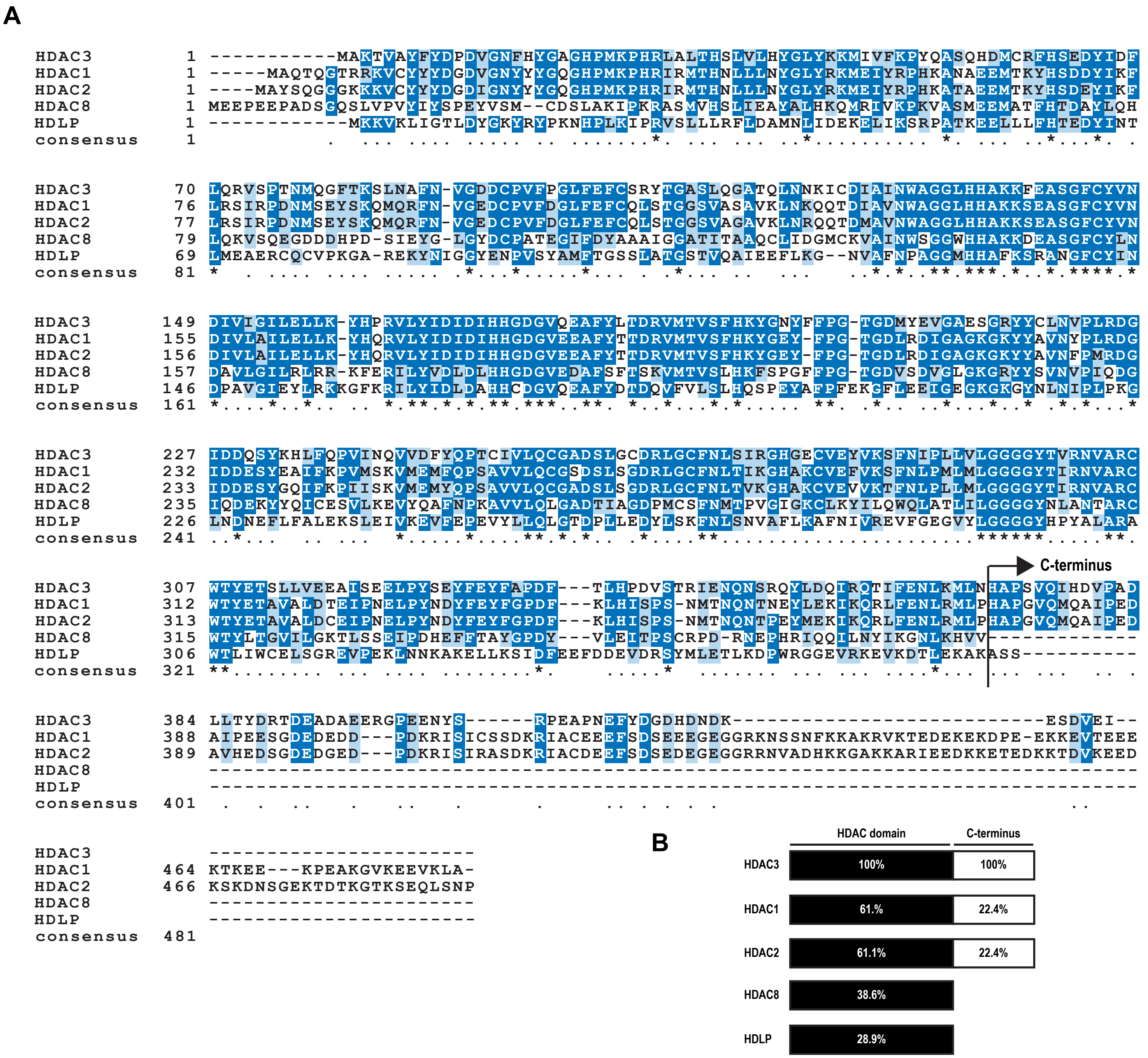

Fig. S1. Sequence alignment and homology comparison of Class I HDACs and HDLP. (**A**) Multiple sequence alignment of human Class I HDACs and HDLP. The location of the C-terminal regions of HDAC1, HDAC2 and HDAC3 is indicated by the right angle arrow. Identical amino acids in different HDACs are highlighted in dark blue. Non-identical but conserved amino acids are highlighted in light blue. (**B**) Comparison of sequence homologies of the HDAC domains and the C-terminal regions. The percentages of identical amino acids of HDAC3 with other Class I HDACs or HDLP in HDAC and C-terminal domains are shown. The alignment was performed using the Clustal Omega software (Sievers F, Wilm A, Dineen D, Gibson TJ, Karplus K, Li W, Lopez R, McWilliam H, Remmert M, Söding J, Thompson JD, Higgins DG. Fast, scalable generation of high-quality protein multiple sequence alignments using Clustal Omega. Mol Syst Biol. 7:539, 2011).

Figure S1

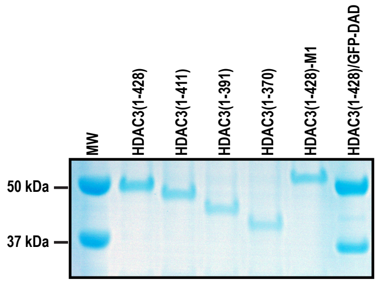

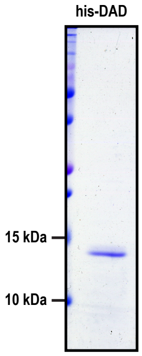

Fig. S2. Recombinant HDAC3, DAD and HDAC3/DAD complex. The images show Coomassie blue staining of recombinant HDAC3(1-428), HDAC3(1-411), HDAC3(1-391), HDAC3(1-370), HDAC3(1-428)-M1, HDAC3/GFP-DAD complex (Left) and His-DAD (Right). All proteins were purified from baculovirus-infected Sf9 cells.

Figure S2

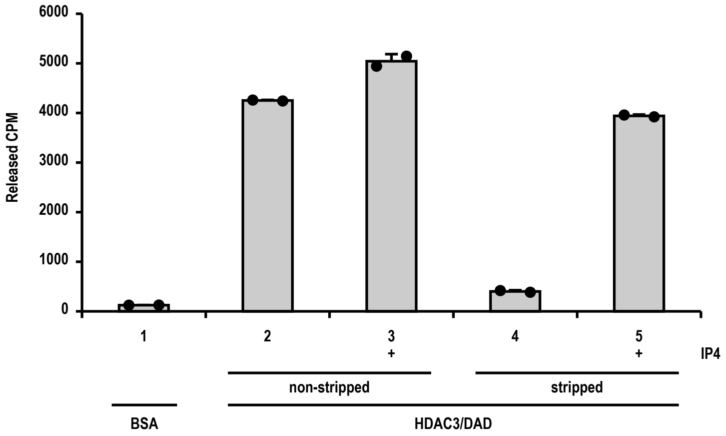

Fig. S3. Liquid scintillation HDAC assays showing the effects of stripping and adding back IP4 on the deacetylase activity of the HDAC3/DAD complex reconstituted in Sf9 cells. The Sf9 insect cells were co-infected with baculoviruses expressing FLAG-tagged HDAC3 and His-tagged DAD. The infected cells were then used for affinity purification of the HDAC3/DAD complex by immunoprecipitating with anti-FLAG antibody followed by elution with the FLAG peptide. The resulting HDAC3/DAD complex, designated as “non-stripped complex”, was then subject to high-salt wash to dissociate IP4 from the complex, resulting in the “stripped” version of the complex. The results show mean + standard deviation from replicated samples (n=2).

Figure S3

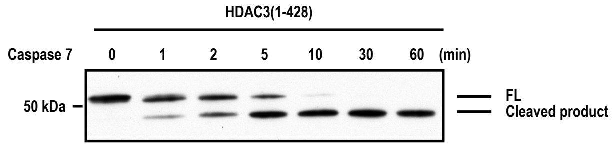

Fig. S4. Free HDAC3 was treated with Caspase 7 for different times as indicated at the top of the gel image, followed by Western Blot analysis using the HDAC3 N-19 antibody.

Figure S4

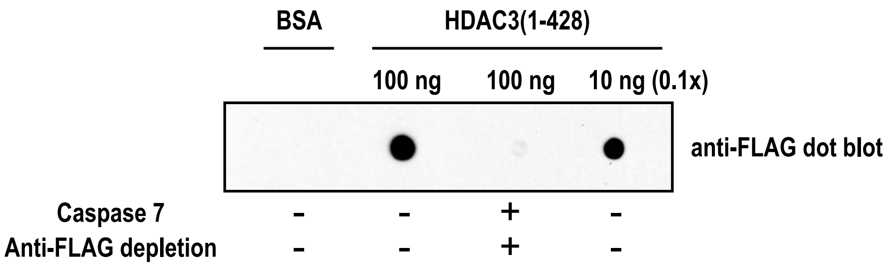

Fig. S5. Removal of the C-terminus upon cleavage of free HDAC3 (1-428) by Caspase 7 via anti-FLAG depletion. In the lane marked with "+", free HDAC3 was cleaved with Caspase 7. After cleavage, the reaction mixture was incubated with the anti-FLAG M2 agarose followed by centrifugation to deplete the released C-terminal tail, which is FLAG-tagged. The supernatant was recovered and subject to dot blot analysis using the anti-FLAG antibody, along with other untreated BSA,100 ng HDAC3(1-428) and 10 ng HDAC3(1-428) samples indicated on the top.

Figure S5

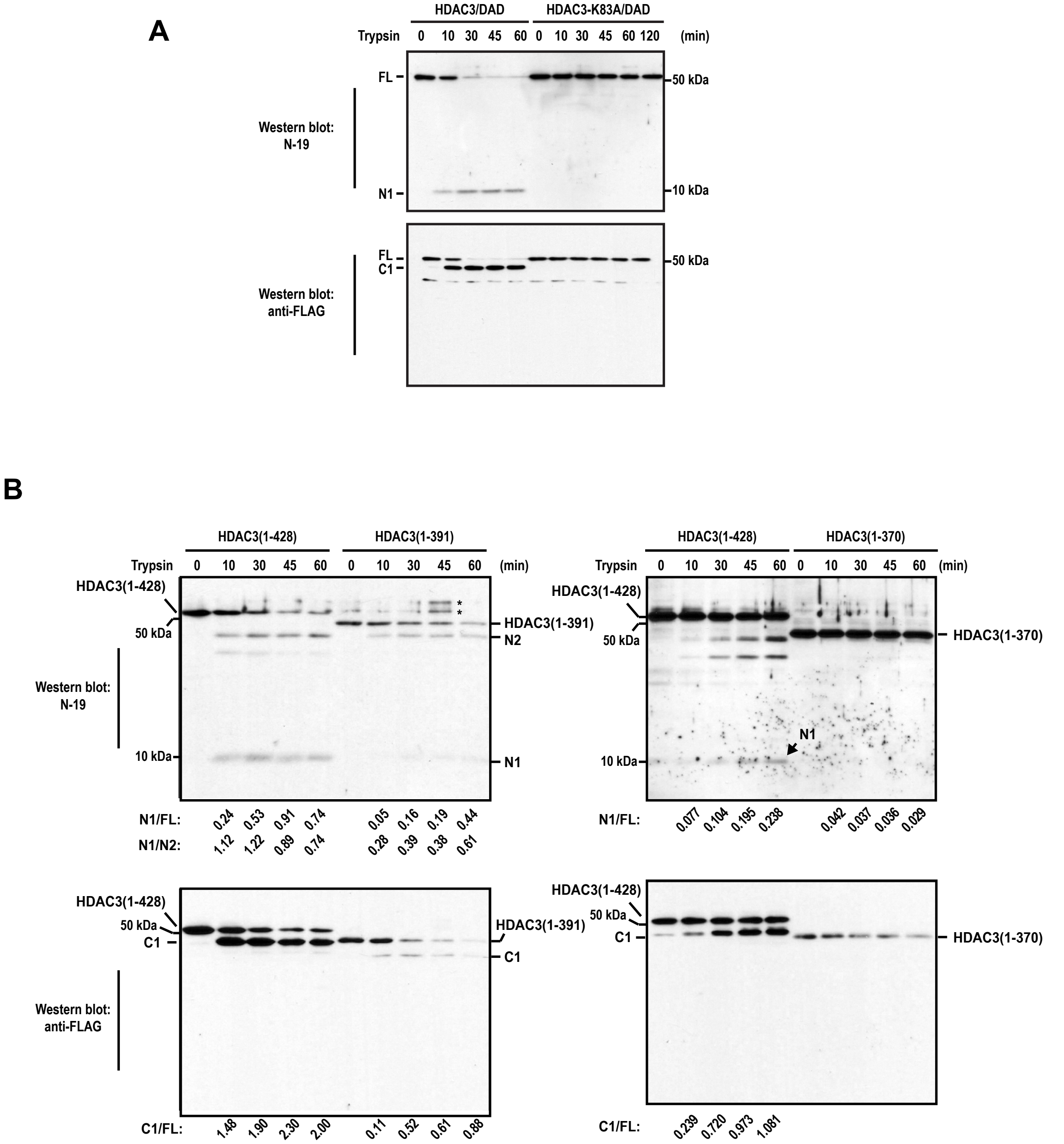

Fig. S6. (A) Trypsin cleaves the N-terminus at K83. N1 and C1 fragments were absent in the trypsin cleavage product of HDAC3-K83A/DAD. **(B) Partial trypsin digestion analyses of FL HDAC3(1-428), HDAC3(1-391) and HDAC3(1-370).** The top panels shows Western blot analysis using N-19 antibody. The bottom panel shows Western blot analysis using anti-FLAG antibody. The experiments were performed and analyzed as in Fig. 2C. The asterisks in the left top panel denote non-specific bands detected by N-19, which were not detected by the anti-FLAG antibody shown at the bottom.

Figure S6

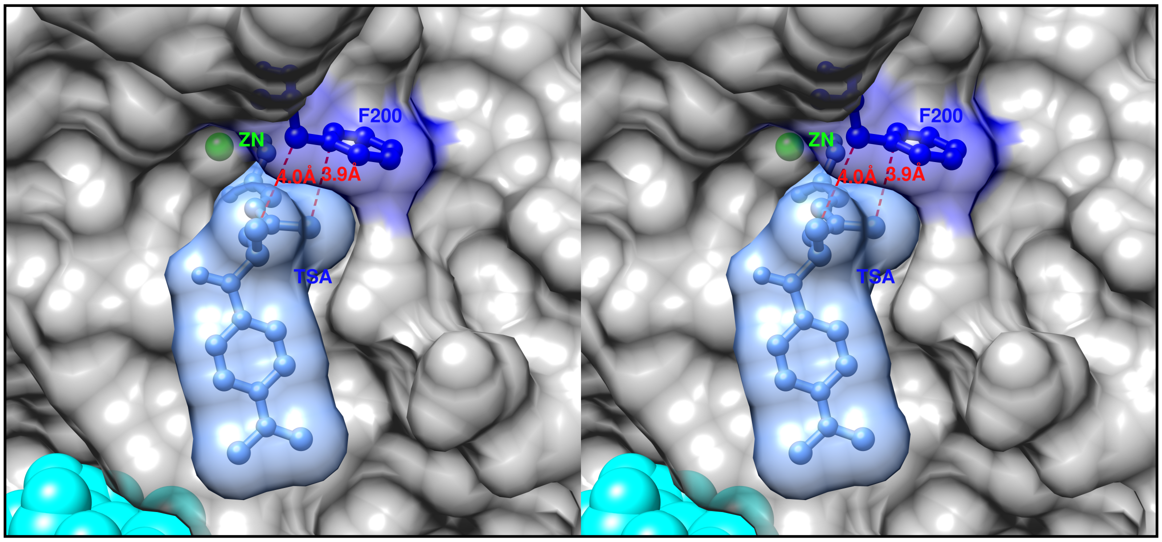

Fig. S7. Wall-eye (or relaxed) stereo images showing that Phe 200 is within contact distance of TSA occupying the substrate pocket. The structure of HDAC3 (PDB# 4A69) was aligned to the structure of TSA/HDLP (PDB# 1C3R) using the UCSF Chimera software. Only the HDAC3 and TSA structures were shown in the figure. Dashed red-colored lines and distances indicate direct contact between the two carbon atoms of the aromatic ring of Phe 200 and C14 and C15 of the side chain of TSA, as detected by the UCSF Chimera software (Pettersen EF, Goddard TD, Huang CC, Couch GS, Greenblatt DM, Meng EC, Ferrin TE. UCSF Chimera--a visualization system for exploratory research and analysis. J Comput Chem. 25(13):1605-12, 2004).

Figure S7

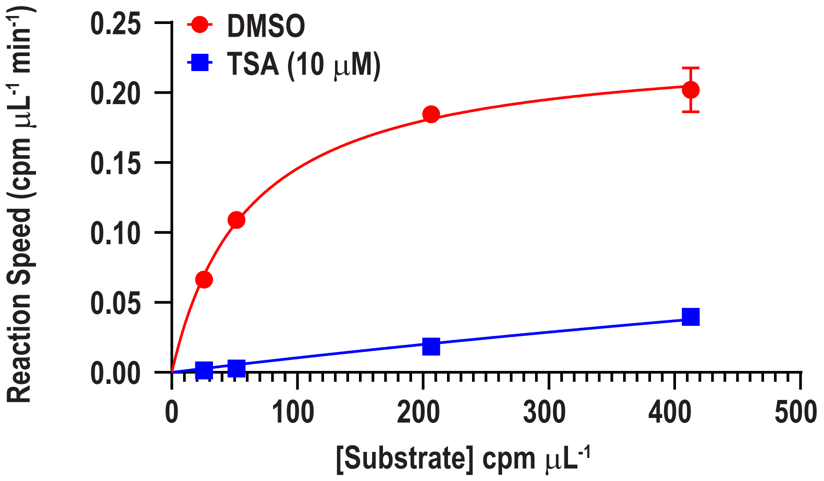

	DMSO	TSA
K_m (cpm μL^{-1})<br>(95% CI)	61.2 ± 4.5<br>(52.3 to 71.7)	
V_{max} (cpm μL^{-1} min^{-1})<br>(95% CI)	0.235 ± 0.005<br>(0.224 to 0.247)	
K_i (μM)<br>(95% CI)		0.293 ± 0.035<br>(0.224 to 0.373)
K_m^{app} (cpm μL^{-1})<br>(95% CI)		2152 ± 224<br>(1744 to 2742)
V_{max}/K_m (K_m^{app}) (95% CI)<br>(slope at low [substrate])	0.00384 ± 0.00022<br>(0.00340 to 0.00434)	0.000109 ± 0.000011<br>(8.62e-005 to 0.000134)

Fig. S8. Michaelis-Menten analysis of free HDAC3 in the absence and presence of TSA. The reaction was performed as described in the Experimental Procedures. The “cpm” refers to “counts per minute” of the radiolabeled substrate (^{3}H-acetyl-histone) used in the assay. Due to unknown molar concentrations of individually acetylated lysines in the histone octamer, “cpm” was used as part of the unit of the substrate, K_m and V_{max}. Non-linear fitting was performed to compare different models based on the Michaelis-Menten kinetics using the Graphpad Prism Program (Version 9.2.0). Compared to noncompetitive inhibition and uncompetitive inhibition, competitive inhibition was found to be the best model as indicated by the Goodness of Fit, and its results (± the standard error) and the 95% confidence interval (CI) were shown in the bottom panel. Michaelis-Menten analysis using only the DMSO data showed the same K_m and V_{max} as the competitive inhibition. Michaelis-Menten analysis using only the TSA data could not reach stable K_m and V_{max} values due to high apparent K_m (K_m^{app}) in the presence of TSA, which is estimated to be ~ 2152. HDAC3 was used at 40 ng. TSA was added at 10 μM.

Figure S8
